# The Systematic Analyses of RING Finger Gene Signature for Predicting the Prognosis of Patients with Hepatocellular Carcinoma

**DOI:** 10.1155/2022/2466006

**Published:** 2022-09-26

**Authors:** Chunfeng Zhang, Yang Yang, Kun Wang, Muhua Chen, Min Lu, Chenyu Hu, Xiaojuan Du, Baocai Xing, Xiaofeng Liu

**Affiliations:** ^1^Department of Medical Genetics, School of Basic Medical Sciences, Peking University Health Science Center, Beijing 100191, China; ^2^Key Laboratory of Carcinogenesis and Translational Research (Ministry of Education), Hepatopancreatobiliary Surgery Department I, Peking University Cancer Hospital & Institute, Beijing 100142, China; ^3^Department of Pathology, School of Basic Medical Sciences, Peking University Health Science Center, Beijing 100191, China; ^4^Department of Cell Biology, School of Basic Medical Sciences, Peking University Health Science Center, Beijing 100191, China

## Abstract

RING finger (RNF) proteins are frequently dysregulated in human malignancies and are tightly associated with tumorigenesis. However, the expression profiles of RNF genes in hepatocellular carcinoma (HCC) and their relations with prognosis remain undetermined. Here, we aimed at constructing a prognostic model according to RNF genes for forecasting the outcomes of HCC patients using the data from The Cancer Genome Atlas (TCGA) program. We collected HCC datasets to validate the values of our model in predicting prognosis of HCC patients from International Cancer Genome Consortium (ICGC) platform. Then, functional experiments were carried out to explore the roles of the representative RNF in HCC progression. A total of 107 differentially expressed RNFs were obtained between TCGA-HCC tumor and normal tissues. After comprehensive evaluation, a prognostic signature composed of 11 RNFs (RNF220, RNF25, TRIM25, BMI1, RNF216P1, RNF115, RNF2, TRAIP, RNF157, RNF145, and RNF19B) was constructed based on TCGA cohort. Then, the Kaplan-Meier (KM) curves and the receiver operating characteristic curve (ROC) were employed to evaluate predictive power of the prognostic model in testing cohort (TCGA) and validation cohort (ICGC). The KM and ROC curves illustrated the good predictive power in testing and validation cohort. The areas under the ROC curve are 0.77 and 0.76 in these two cohorts, respectively. Among the prognostic signature genes, BMI1 was selected as a representative for functional study. We found that BMI1 protein level was significantly upregulated in HCC tissues. Moreover, the inhibitor of BMI1, PTC-209, displayed an excellent anti-HCC effect *in vitro*. Enrichment analysis of BMI1 downstream targets showed that BMI1 might be involved in tumor immunotherapy. Together, our overall analyses revealed that the 11-RNFs prognostic signature might provide us latent chances for evaluating HCC prognosis and developing novel HCC therapy.

## 1. Introduction

Hepatocellular carcinoma (HCC) is one of the most common malignancies worldwide. HCC resulted in approximately 781,000 deaths worldwide in 2018, ranking as the fourth leading cause of cancer-related death according to the assessment by GLOBOCAN [1, 2]. Despite a series of treatment strategies for HCC have been developed, the overall outcome of HCC patients is poor. In the current, the optimal therapy is curative resection for HCC at early stage, while lots of cases are diagnosed in advanced stage with missing surgical timing [3]. Upon tumor progression, the accumulated somatic DNA alterations constantly help cancer cells gain malignant behaviors [4, 5]. Due to the wide application of high-throughput sequencing method, researchers gain the chance to globally understand the molecular changes in hepatic cancer cells and establish molecular model for evaluating the status of HCC [6]. It has been found that HCC patients with the same clinical stage own specific molecular subtypes and gene signatures [7]. This further supports the possibility to predict HCC patients' outcomes at molecular level. The establishment of prognosis-related molecular model and the discovery of new therapeutic targets for HCC will be helpful for improving the survival rates of HCC patients.

RING finger (RNF) proteins comprise a large family of proteins which play pivotal roles in protein ubiquitination. Ubiquitination is mainly involved in mediating protein degradation, which in turn regulates cellular activities [8, 9]. It has been reported that ubiquitination participates in lots of intracellular biological processes, such as affecting DNA damage repair, modulating cell metabolism, regulating cell death, and altering therapeutic effect [10]. Ubiquitination is defined as a multistep biochemical reaction, which transfers ubiquitin molecules to the substrates. The indispensable enzymes in this reaction include ubiquitin-activating enzyme (E1), ubiquitin-binding enzyme (E2), and ubiquitin ligase (E3) [11]. Among the enzymes, E3 ligases are responsible for specifically recognizing the substrates and transferring ubiquitin to substrates. In eukaryotes, hundreds of E3 ligases have been identified. Generally, E3s mainly fall into three classes based on the conserved domains for ligase activity, namely HECT, RING finger, and U-box [12]. RNF proteins belong to RING finger E3 harboring RINF finger domain [13]. Dysfunction of RNFs leads to intricate alterations of the transcriptome and proteome in tumor cells, further causing changes in cellular activities, including cell growth, proliferation, apoptosis, migration, and invasion [14, 15]. Increasing number of studies have reported that some RNFs are exceptionally expressed in human cancers and are associated with poor prognosis of patients [16, 17], indicating that the certain RNFs might be latent targets for cancer diagnosis and therapy.

Recent studies have found that some RNF proteins play momentous roles in development and progression of HCC. RNF147, also named TRIM25, enhances the HCC cell survival upon cellular stress by targeting Keap1-Nrf2 pathway [18]. RNF2 promotes ubiquitination of SIK1 in HCC cells and promotes cell growth [19]. The overexpression of RNF40, as an E3 ligase of H2B ubiquitination, indicates poor prognosis of HCC patients [20, 21]. These studies indicate that some RNFs are tightly associated with the progression of HCC. The aberrant expression and function of these RNFs offer us new chances for developing inhibitors of HCC. However, the prognostic roles of RNFs in HCC remain undetermined and this urges us to explore the comprehensive roles of RNF-related genes in HCC.

In the present study, we collected RNA-sequencing data of HCC samples from TCGA and ICGC platforms. After evaluating transcriptomic alterations of RNF genes between HCC and nontumorous tissues, we constructed a risk score model with 11 prognostic RNFs. Moreover, BMI1 was selected as the representative to explore its roles in HCC through functional experiments. Ultimately, we uncovered an RNF-related signature related to the pathogenesis of HCC, which might be applied as latent prognosis-related biomarkers and drug targets for HCC.

## 2. Materials and Methods

### 2.1. Patient Samples and Immunoblot

In total, 18 paired HCC tissues and noncancerous tissues were obtained from the Peking University Cancer Hospital. The study was approved by the ethics committee of the Peking University Cancer Hospital. Western blot was carried out according to the previous reports [22, 23]. Anti-BMI1 antibody (A0211) and Anti-*β*-actin antibody (AC026-100) were purchased from Abclonal Technology (China). PTC-209 (S7372-PTC-209) was purchased from Selleck Chemicals (USA).

### 2.2. Cell Culture

HepG2, SMMC-7721, or Huh7 cell lines were purchased from the National Infrastructure of Cell Line Resource (NSTI, China). We cultured cells using DMEM or RPMI 1640 medium added with 10% fetal bovine serum. After passage or indicated treatment, cells were cultured in a humidified chamber in 5% CO_2_ at 37°C.

### 2.3. Cell Proliferation Evaluation

Cell proliferation was analyzed using MTS kit (Promega, USA). Briefly, cells were digested and seeded into the 96-well plate. Then, the drugs were added to the cells as indicated. Cell number was determined for each day by MTS assay according to the manufacturer' protocol.

### 2.4. Colony Formation Experiment

Colony formation assay was carried out according to previously published protocol [24]. Briefly, cells were treated with the indicated concentrations of PTC-209 and subsequently seeded into 6-well plate. Fourteen days later, the colonies were fixed with paraformaldehyde and stained with 0.1% crystal violet (Beyotime, China). The visible colonies were counted using ImageJ software.

### 2.5. Data Collection and Analysis

The RNA sequencing data and the related clinical data of HCC were obtained from TCGA website (https://portal.gdc.cancer.gov/). The RNF gene set including 227 genes was obtained from GEPIA website [25]. We preprocessed the raw data using Limma package in R software. Then, we collected the RNFs expression profile from LIHC (Liver Hepatocellular Carcinoma) dataset including 374 tumorous and 50 normal samples for the following analyses. The differentially expressed RNFs were identified using the criteria: |log_2_FC ≥ 1| and FDR < 0.05. The “pheatmap” package was employed for unsupervised clustering analysis in R software. The validation dataset of gene expression and clinical trait data (the Liver Cancer-RIKEN JP) was collected from the ICGC database (https://dcc.icgc.org/). GSE97172 dataset was downloaded from GEO database (https://www.ncbi.nlm.nih.gov/gds/). Similarly, the raw data was preprocessed using R software.

### 2.6. KEGG (Kyoto Encyclopedia of Genes and Genomes) Pathway and GO (Gene Ontology) Enrichment Analyses

Briefly, GO and KEGG pathway enrichment analyses were carried out using the DAVID platform (https://david.ncifcrf.gov/) [26]. The GO analysis terms contain molecular function (MF), cellular component (CC), and biological process (BP). The GO enrichment results and KEGG pathways were visualized through the “GOplot package” in R software. We used *p* < 0.05 as the threshold for statistical significance.

### 2.7. PPI (Protein-Protein Interaction) Network Construction and Key Modules Identification

The PPI network of the differentially expressed genes was established through the STRING database (http://www.string-db.org/) [27]. The results gained by STRING database were further analyzed and visualized using Cytoscape 3.7.1 software. The key modules were discovered using Molecular Complex Detection (MCODE) plug-in based on MCODE score and node counts.

### 2.8. RNFs-Based Prognostic Model Construction

We performed univariate Cox regression analysis to identify the prognosis-related RNF genes using the “survival” package in R software. *p* < 0.05 was used as the statistically significant. Lasso Cox regression analyses were further performed for uncovering prognostic signatures using the “glmnet” package in R software. The risk score for each sample was calculated according to RNFs expression (Expi) and coefficient value (*β*i): Risk score = exp (0.20^∗^RNF220 + 0.33^∗^RNF25 + 0.04^∗^TRIM25 + 0.11^∗^BMI1 + 0.057^∗^RNF216P1 + 0.007^∗^RNF115 + 0.019^∗^TRAIP + 0.12^∗^RNF2 + 0.039^∗^RNF157 + 0.14^∗^RNF145 + 0.21^∗^RNF19B).

According to the median of risk score values, HCC patients were divided into high-risk and low-risk groups for the subsequent analyses. The “survival” package was utilized to calculate the differences of overall survival (OS) time between the two groups. Besides, the ROC curve analysis was also performed to evaluate the prognostic capability of this model using the “survivalROC” package in R software.

### 2.9. Genomic Analysis and Drug Prediction for Prognostic RNFs

The mutation of RNF genes was analyzed through cBioPortal (https://www.cbioportal.org/), which is a public platform used for analyzing and visualizing the cancer genomics datasets. All data for RNFs-related drugs were analyzed through PharmacoDB (https://pharmacodb.pmgenomics.ca/) [28].

### 2.10. Detection of the Risk Genes in Protein Level

The protein expression level of these risk genes was evaluated through the Human Protein Atlas (HPA) database for further verifying the transcriptional level of the related genes (https://www.proteinatlas.org/).

### 2.11. Statistical Analyses

Statistical analyses were conducted using the SPSS software (Inc., Chicago, IL) or R software. Univariate and multivariate Cox analyses were utilized to identify independent prognostic factors. The overall survival (OS) and disease-free survival (DFS) curves were calculated using Kaplan-Meier analysis, and the statistical significance between different groups was calculated by log-rank test. A Student's *t*-test was used to calculate the significance between two groups of the indicated samples. *p* < 0.05 was used as statistically significant.

## 3. Results

### 3.1. The Differently Expressed RNFs Were Identified between Hepatic Normal and Tumorous Tissues

**Figure 1:**
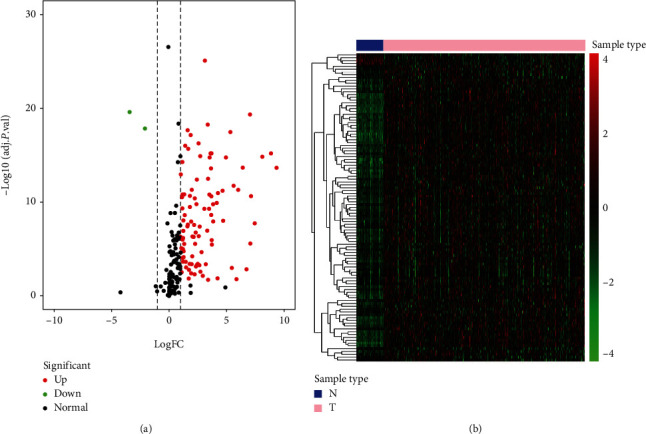
The differentially expressed RNFs were identified between hepatic normal and tumor tissues. (a) Volcano plot displaying RNFs. The upregulated RNF is shown in red and downregulated RNF is shown in green. Black means no difference. (b) Hierarchical clustering of differentially expressed RNFs is presented. The columns mean samples and the rows indicate RNFs. The green means downregulation while the red means upregulation.

To study the roles of RNFs in HCC, we downloaded the transcriptomic file from the TCGA-LIHC dataset consisting of 50 normal tissue samples and 374 HCC samples. Then, we screened the differently expressed RNFs using the cut-off values of FDR < 0.05 and |log_2_FC| ≥ 1. Totally, 105 upregulated and 2 downregulated RNF genes were uncovered (Figure 1(a)). The expression profile of these identified RNFs is described in Figure 1(b).

### 3.2. GO and KEGG Pathway Analyses Were Performed with the Differently Expressed RNFs

**Figure 2:**
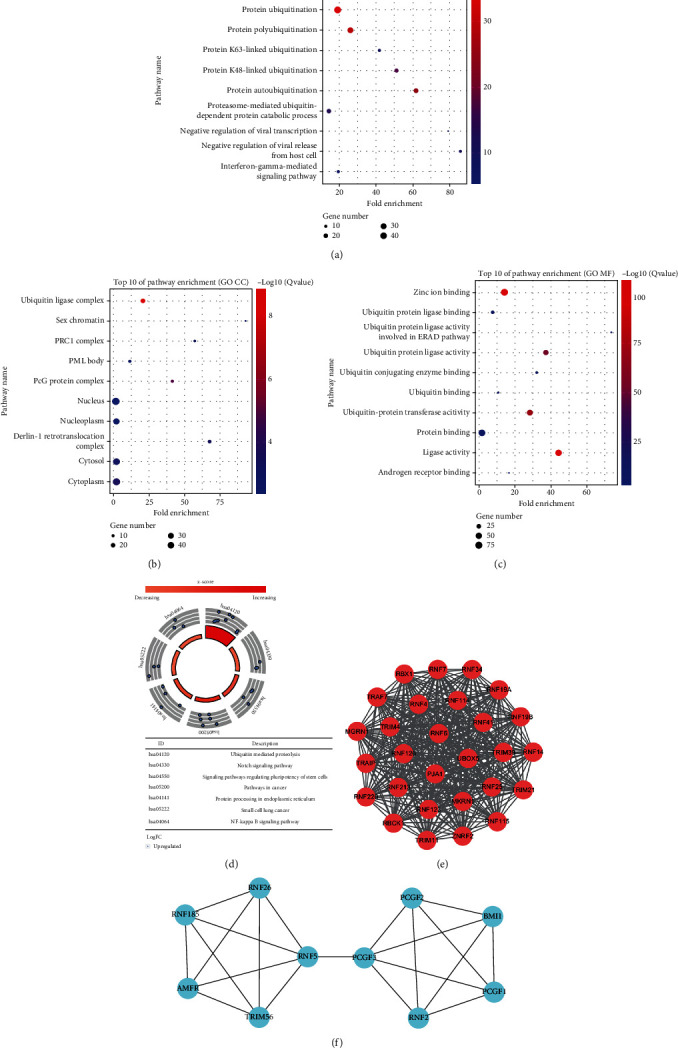
GO enrichment, KEGG pathway, and PPI network analyses of differentially expressed RNFs. (a–c) The top 10 items enriched GOBP (a), GOCC (b), and GOMF (c) in GO analyses. (d) The top seven enriched pathways are shown in KEGG analysis. (e) Module 1.MCODE score = 28.00, Nodes = 28, Edges = 378. (f) Module 2.MCODE score = 4.67, Nodes = 10, Edges = 21.

To analyze the function of these identified RNFs, we conducted GO enrichment and KEGG pathway analyses. GO analyses categorized RNFs into three groups including GOBP, GOCC, and GOMF. The top 10 enriched GOBP, GOCC, and GOMF are presented in Figures 2(a)–2(c), respectively. Based on the GO analyses, differently expressed RNFs were mainly involved in protein polyubiquitination, ubiquitin ligase complex, and zinc-ion binding pathways. Furthermore, KEGG analysis suggest that the top 5 enriched pathways were “ubiquitin mediated proteolysis”, “Notch signaling pathway”, “signaling pathways regulating pluripotency of stem cells”, “pathways in cancer”, and “protein processing in endoplasmic reticulum” (Figure 2(d)). These pathways are tightly associated with protein ubiquitination and tumorigenesis. Thus, these enrichment analyses of these differently expressed RNFs in HCC revealed that the alterations of ubiquitination network caused by these RNFs contribute to HCC development and progression.

### 3.3. The PPI Network Was Constructed Using the Differently Expressed RNFs

Next, we explored the critical protein association networks of these 107 RNFs. STRING platform were utilized to establish PPI network for exploring the interactions among these RNFs. We obtained 103 nodes and 548 edges using a *p* value of PPI concentration<1.0e-16 as selection criteria. The top two enriched clusters in the PPI network were identified using the Cytoscape with MCODE plug-in (Figures 2(e) and 2(f)). The function of each cluster was next analyzed by pathway enrichment analysis. The results revealed that Module 1 was mainly involved in ubiquitin-mediated proteolysis. Module 2 mainly participated in regulating pluripotency of stem cells and ER (endoplasmic reticulum)-related protein processing. Thus, the PPI analysis confirmed that RNFs play an important role in protein ubiquitination, which is tightly associated with HCC progression.

### 3.4. Recognition of Prognosis-Related RNFs and Establishment of Prognostic Model in HCC

**Figure 3:**
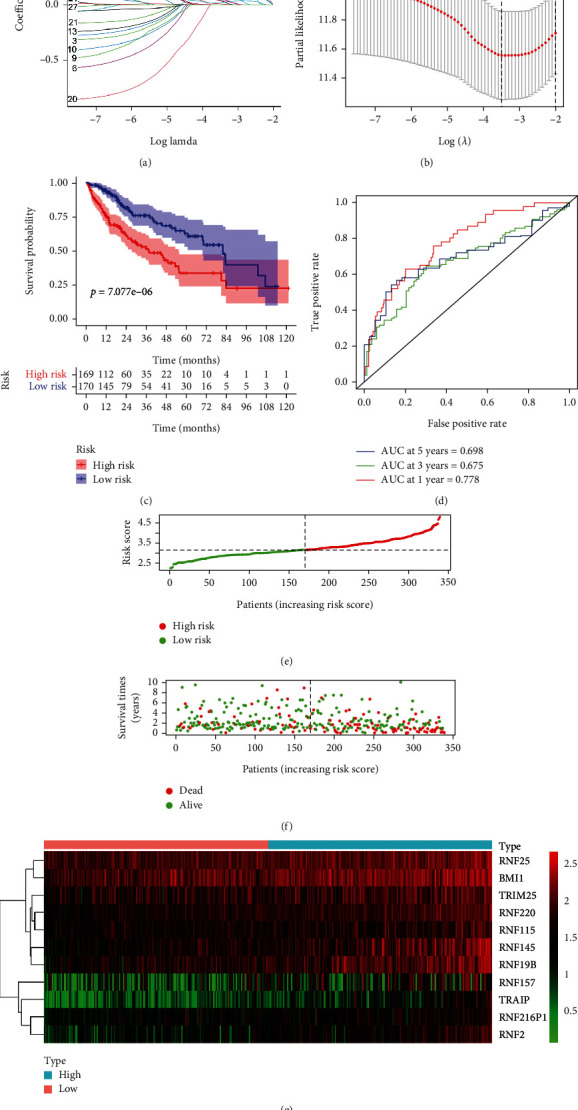
The risk score model was established based on prognostic RNFs using the TCGA HCC cohort. (a) Each curve indicates an *RNF* gene and the best lambda was computed to minimize mean cross-validated error. (b) The proportional hazards model was employed for cross-validation to select tuning parameter. (c) Kaplan-Meier analysis of TCGA-LIHC patients based on risk score. (d) Time-dependent ROC curves were established to estimate OS according to the risk score. (e) HCC patients were subdivided into high-risk and low-risk groups using the median of risk scores as cut-off. (f) Scatter plots displayed the associations of risk score status with survival outcome in TCGA HCC patients. (g) The heat map displayed the expression levels of prognostic RNFs in each HCC patient from TCGA cohort. The red represents increased expression and the green indicates decreased expression.

To explore the prognostic value of the differently expressed RNFs, the univariate Cox regression analyses were conducted using corresponding TCGA clinical data. As shown in Supplementary Figure S1, 29 candidate RNFs were found to be associated with the overall survival (OS) of HCC patients. Moreover, we performed Lasso Cox regression analysis to select the prognostic RNFs for constructing prognostic model (Figures 3(a) and 3(b)). According to the integrated prognostic relevance, 11 RNF genes (RNF220, RNF25, TRIM25, BMI1, RNF216P1, RNF115, RNF2, TRAIP, RNF157, RNF145, and RNF19B) were selected to construct a risk score model. We calculated the risk score for each patient according to expression values of 11 RNFs as follows: Risk score = exp(0.20^∗^RNF220 + 0.33^∗^RNF25 + 0.04^∗^TRIM25 + 0.11^∗^BMI1 + 0.057^∗^RNF216P1 + 0.007^∗^RNF115 + 0.019^∗^TRAIP + 0.12^∗^RNF2 + 0.039^∗^RNF157 + 0.14^∗^RNF145 + 0.21^∗^RNF19B). According to results from Lasso-penalized Cox regression, all these RNFs had positive coefficients and acted as independent prognostic factors for OS of the patients with HCC.

Based on the median of risk score values, the patients with HCC were divided into low-risk and high-risk groups. Compared with the patients in the low-risk, HCC patients in high-risk group had a worse outcome by OS analysis in TCGA cohort (Figure 3(c)). In addition, we employed the ROC analysis to evaluate the prognostic ability of the risk model. As shown in Figure 3(d), the areas under the ROC curve (AUC) of our model were 0.778, 0.675 and 0.698 at 1 year, 3 years, and 5 years, respectively, using the data of TCGA cohort. This result suggests that the risk score model is more accurate in the short-term follow-up. Moreover, the risk scores of these patients were ranked and exhibited according to risk score (Figure 3(e)). The survival status of each HCC patient in TCGA-LIHC cohort was shown in Figure 3(f). Consistently, there were shorter OS time and higher mortality rates in the patients with high-risk than those in the low-risk (Figure 3(f)). These data confirmed the utility of our risk score model in evaluating the prognosis of HCC patients. Additionally, the expression profiles of these prognosis-related RNFs between the two groups were presented in Figure 3(g). The result revealed that all the prognostic RNFs were upregulated in the HCC patients with high-risk.

### 3.5. Independent Prognostic Value of the Risk Score Model Was Analyzed in HCC

**Figure 4:**
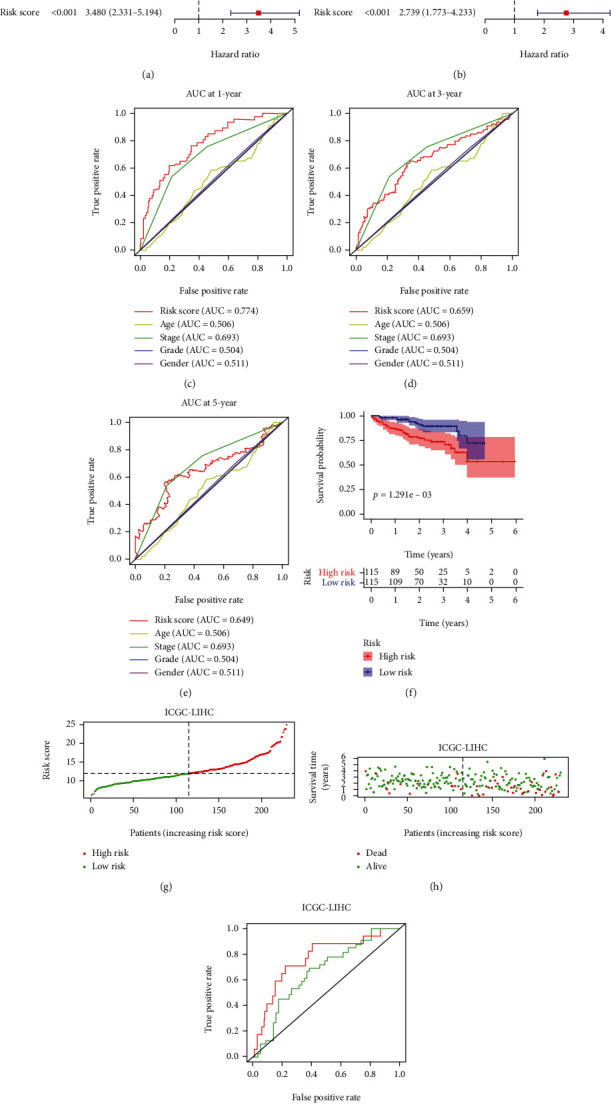
Validation of the reliability of the risk score model. (a) Univariate Cox regression analysis of the indicators. (b) Multivariate Cox regression analysis of the indicators. (c–e) Time-dependent ROC curves were used to analyze the predicting ability of the indicated factors in the TCGA HCC cohort. (f) Kaplan-Meier curve of ICGC patients was analyzed according to the risk score. (g) ICGC-LIHC patients were divided into high-risk and low-risk groups based on the median of risk score. (h) Scatter plots displayed the relationships of risk score status with the survival outcome in the ICGC HCC patients. (i) Time-dependent ROC curves were calculated according to risk scores in the ICGC HCC cohort.

Subsequently, we performed univariate Cox regression analysis to explore the relations of clinic-pathological characteristics and the risk score with prognosis in the TCGA-LIHC patients. As shown in Figure 4(a), tumor stage and risk score were tightly related to the overall survival. The following multivariate Cox regression analysis further confirmed that risk score and TNM stage were two independent prognostic factors of survival for patients with HCC (Figure 4(b)). Next, we compared the AUC values of these clinical factors at 1-year, 3-year, or 5-year. The results indicated that our model more precisely forecasted 1-year OS rate compared to TNM stage (Figures 4(c)–4(e)).

### 3.6. The Prognostic Signature Was Validated for OS Prediction in ICGC Cohort

For confirming the predictive power of our model, HCC patients with clinical information from the ICGC were enrolled as a validation cohort. Based on the expression level of the 11 RNFs, the risk score of each patient were calculated using the risk score formula. Then, we used the median of risk score values as cut-off and subdivided the patients into high-risk and low-risk groups. We analyzed the difference of survival of these two groups by survival analysis and found that patients with high-risk had a shorter OS compared with the patients with low risk (Figure 4(f)). Then, the risk scores of these HCC patients were ranked and exhibited in Figure 4(g). The survival status of each ICGC-HCC patient was estimated. As shown in Figure 4(h), there were shorter OS time and higher mortality rates in the patients with high-risk than those in the low-risk group. ROC curve was further calculated and the AUC for the OS model at 1-year and 3-year were 0.766 and 0.662, respectively, in ICGC cohort (Figure 4(i)). Together, these data indicated that our risk score model is useful for estimating the outcome of ICGC HCC patients. Together, our risk score model based on RNF gene expression precisely predicts the prognosis of HCC patients.

### 3.7. The Relationships between the Prognosis-Related RNFs and Clinicopathological Features Were Analyzed

**Figure 5:**
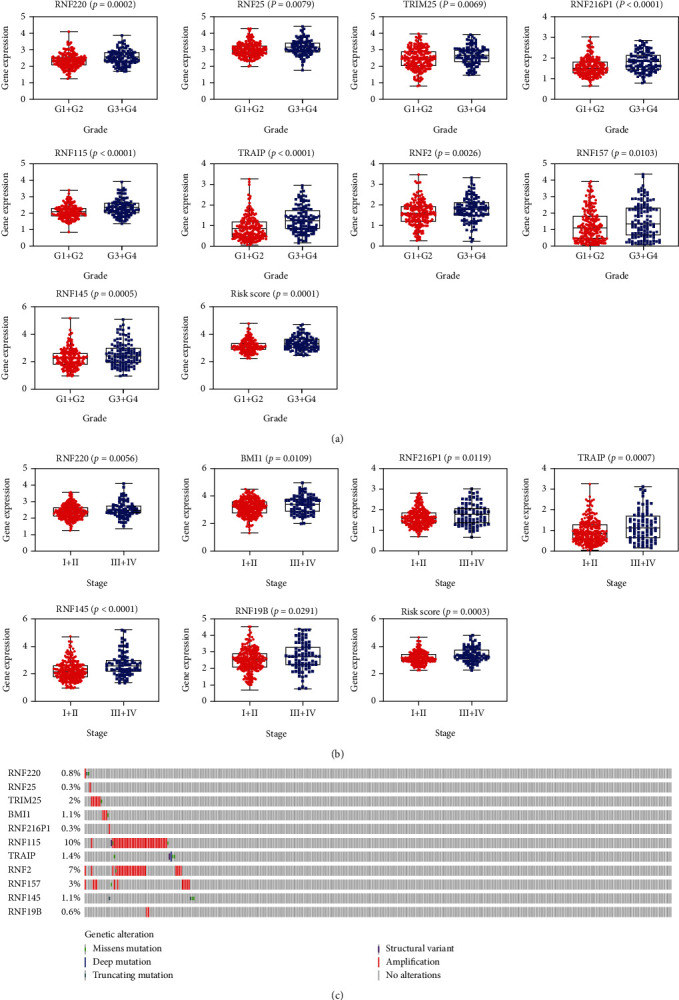
The relevance between prognostic RNFs and clinicopathologic features was analyzed. (a) The association of these prognostic RNFs with tumor grade was evaluated. (b) The relationship between prognostic RNFs and tumor stage was analyzed. (c) Oncoplot for each prognostic *RNF* gene.

Next, we explored the associations of these prognostic RNFs and clinicopathological features, including TNM stage and tumor grade. As shown in Figure 5(a), nine RNFs (RNF220, RNF25, TRIM25, RNF216P1, RNF115, TRAIP, RNF2, RNF157, and RNF145) were found to be upregulated in HCC patients with advanced grade (*p* < 0.05). Moreover, six RNFs were increased in patients with advanced stage, including RNF220, BMI1, RNF216P1, TRAIP, RNF145, and RNF19B (Figure 5(b)). Importantly, the risk scores of patients with advanced grade or stage are much higher, indicating that our prognostic model was associated with both tumor grade and TNM stage.

The genetic changes of these RNFs were further determined using the cBioPortal website. Mainly, the genetic alterations of these RNF genes include truncating mutation, missense mutation, deep deletion, structural variant, and amplification. The top 5 most significantly altered genes are RNF115, RNF2, RNF157, TRIM25, and TRAIP in HCC samples (Figure 5(c)).

### 3.8. The Protein Levels of Prognostic RNFs Expression Were Analyzed through the HPA Database

**Figure 6:**
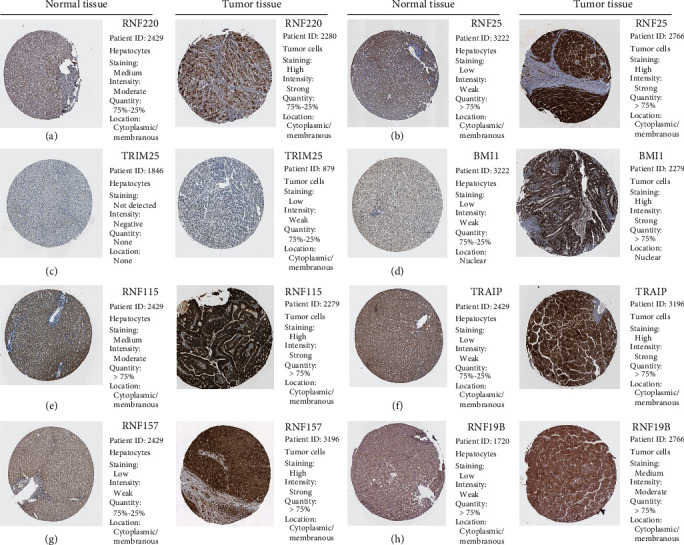
The protein levels of the prognostic RNFs were evaluated in the HPA database. (a–h) IHC staining of prognostic RNF in HCC tumor tissues and normal liver tissues.

We next assessed the protein levels of the 11 prognostic RNFs through the HPA database. We found that eight RNFs including RNF220, RNF25, TRIM25, BMI1, RNF115, TRAIP, RNF157, and RNF19B were overexpressed in HCC cells compared to normal cells by immunohistochemistry (IHC) staining (Figures 6(a)–6(h)). Finally, the staining of RNF2 and RNF145 proteins was missing and needs further analysis.

### 3.9. Functional Study of the Roles of BMI1 in HCC Cells

Due to the prognostic values of the 11 RNFs for HCC patients, we next explored the potential drugs for these RNFs through the PharmacoDB database. Only BMI1 was identified as small molecule targets among these prognostic RNFs. As a core element of polycomb repressive complex 1(PRC1), BMI1 has been found to be associated with various human cancers and become an attractive therapeutic target. One of the specific BMI1 inhibitors, PTC-209, displays high potency in repressing the growth of some types of cancer cells [29, 30]. However, it is unknown whether PTC-209 shows the potential capability in anti-HCC therapy.

**Figure 7:**
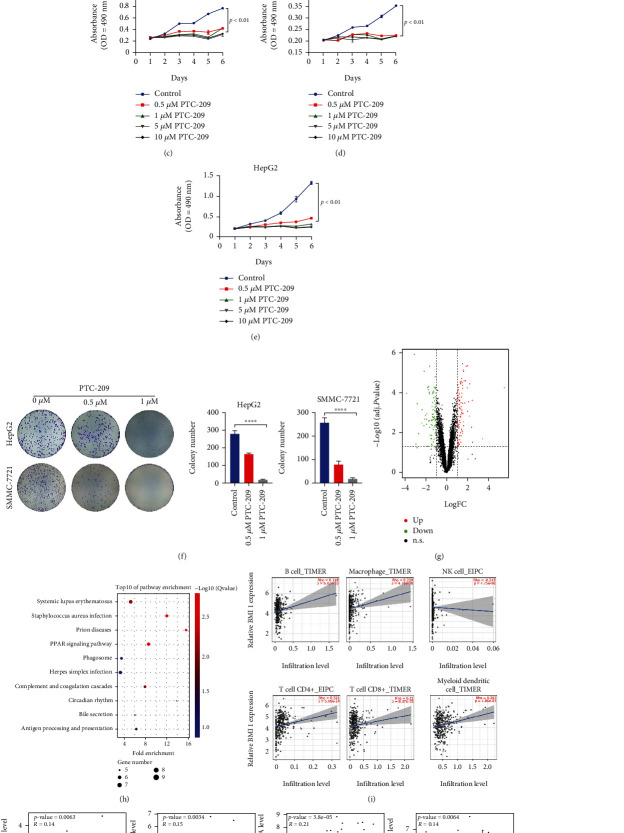
Functional studies of BMI1 in HCC progression. (a) Kaplan-Meier survival analysis for overall survival and disease-free survival of the TCGA-LIHC patients based on BMI1 mRNA level. (b) Western blot analysis of BMI1 expression in 18 individual paired HCC tissues. (c–e) Cell proliferation assay was performed in HCC cells after PTC-209 treatment. (f) Colony formation assay was conducted in HCC cells treated with PTC-209. (^∗∗∗∗^*p* < 0.0001) (g) Volcano plot for DEGs between BMI1 wild-type and BMI1 knockout mice liver tissues using GSE97172 dataset. (h) KEGG pathway analysis for DEGs identified in (g). (i) The correlation of BMI1 expression with immune infiltration level was analyzed in HCC tissues. (j) Coexpression analysis between the expression level of PD-L1, Galectin 9, HVEM, IDO, and BMI1 using TCGA-LIHC data.

Survival analysis using TCGA-LIHC cohort revealed that the high BMI1 expression was associated with shorter OS and DFS in the HCC patients (Figure 7(a)). Moreover, the expression of BMI1 on 18 paired cancerous and matched noncancerous sections of HCC tissues from our center was evaluated by immunoblotting. As shown in Figure 7(b), BMI1 was upregulated in cancerous tissues compared to peritumoral tissues. Importantly, the MTS assay showed that the proliferation of HCC cells was significantly repressed by PTC-209 treatment (Figures 7(c)–7(e)). To further confirm our results, we randomly selected SMMC-7721 and HepG2 cells to perform colony formation assay. Consistently, colony formation of HCC cells was significantly decreased upon PTC-209 treatment (Figure 7(f)). Together, these data indicated that PTC-209 inhibits HCC cell proliferation and growth *in vitro*.

To explore the mechanisms underlying BMI1-induced HCC progression, we analyzed the differentially expressed genes in liver tissues from BMI1-knockout mice (GSE97172). As shown in Figure 7(g), 624 genes were found to be differentially expressed. KEGG analysis suggested that immune system-related pathways were significantly altered upon BMI1 loss (Figure 7(h)). These results indicated that BMI1 might be involved in regulating tumor immune microenvironment. Tumor-infiltrating lymphocytes can be used as an independent indicator of the survival and sentinel lymph node status in human cancers [31]. Therefore, we analyzed whether BMI1 expression was correlated with the infiltration of immune cells in HCC using TIMER website. The results show that BMI1 expression has positive correlations with CD8^+^ T cell, CD4^+^ T cell, neutrophil, B cell, and dendritic cell infiltration levels while BMI1 expression is negatively associated with natural killer cell infiltration level (Figure 7(i)). We also assessed the correlation between BMI1 expression and immune checkpoint-related molecules, including PD-L1, Galectin 9, HVEM, and IDO1, all of which are important marker genes for cancer immune therapy [32]. As shown in Figure 7(j), there were positive correlations between BMI1 expression level and the expression levels of these four marker genes. Together, these data suggest that BMI1 might be a novel target in HCC immunotherapy.

## 4. Discussion

Metastasis and recurrence after resection are common for HCC, which causes treatment failure and cancer-related death [33–35]. Development of prognostic assessment system will be favorable for follow-up after treatment, in order to attenuate tumor progression caused by metastasis or relapse, especially in patients with high-risk. Clinically, prognostic evaluation includes tumor status, cancer-related symptoms, and liver function of the patient [36]. Recently, with the improvement of high-throughput technologies, it is possible to develop molecular typing for cancer diagnosis and treatment. In the present study, we identified RNF-based molecular biomarkers and constructed a risk score model to forecast outcomes of HCC patients using TCGA-LIHC cohort. We further validated the risk model using ICGC-LIHC dataset. Moreover, we also explored the roles of a typical prognostic RNFs and BMI1, in HCC progression. Recently, some latent biomarkers and therapeutic targets have been identified for HCC by bioinformatics strategies. The eleven RNA-binding proteins (RBPs) were screened to construct a prognostic model for indicating overall outcomes of HCC patients [37]. A four-gene metabolic signature predicting OS for HCC was built [38]. The immune-related gene prognostic signature for HCC was also constructed [39]. Currently, the prognosis model based on the RNFs of HCC has not been reported. Thus, our study determines the prognostic values of RNF genes in HCC and provides a new idea for HCC diagnosis and treatment.

Dysregulation of some RNFs leads to abnormal ubiquitination of the important proteins in tumor cells and drives tumorigenesis, including HCC [40]. Here, we aimed at studying the universal roles of RNF genes in forecasting OS of HCC patients. Through screening differentially expressed genes between HCC and normal tissues, total 107 RNFs were identified as differentially expressed RNFs using TCGA cohort. Then, GO enrichment and KEGG pathway analyses revealed that the differentially expressed RNFs were greatly involved in ubiquitin-mediated proteolysis. We uncovered 29 prognosis-associated candidate RNFs by univariate Cox regression analysis. With Lasso Cox regression, eleven RNFs genes (RNF220, RNF25, TRIM25, BMI1, RNF216P1, RNF115, RNF2, TRAIP, RNF157, RNF145, and RNF19B) were identified to construct a risk score model. We confirmed the stability and reliability of this model using ICGC data as the validation set. The results suggested that the model is accurate for distinguishing HCC patients with different survival outcomes. Univariate and multivariate analyses further confirmed that this prognosis model could independently indicate overall prognosis of patients with HCC. ROC curve also manifested that our model based on the 11 RNFs had a good predictive ability. These results suggest that our risk model might be applied to screen high-risk patients for personalized detection or follow-up.

Among the eleven RNF genes, the majority (RNF220, RNF25, TRIM25, RNF115, BMI1, TRAIP, RNF2, RNF157, RNF145, and RNF19B) have been reported to function in ubiquitination and play roles in tumorigenesis. RNF220 is associated with progression of leukemia or medulloblastoma [41, 42]. RNF25 upregulates gefitinib resistance via promoting ERK reactivation in EGFR-mutant NSCLC cells [43]. TRIM25 takes part in tumor growth, metastasis, and chemoresistance with its ubiquitin ligase activities [18, 44]. RNF115 is correlated with the prognosis of patients with lung adenocarcinoma or invasive breast cancer [45, 46]. As a master regulator of DNA repair, dysfunction of TRAIP is associated with tumor development and progression [47, 48]. Overexpression of RNF2 is positively correlated with progression of many cancers, including HCC, melanoma, pancreatic cancer, and gastric cancer [49].

BMI1 is a core element of the PRC1 complex which mediates gene silencing via monoubiquitination of histone H2A. The polycomb group (PcG) proteins encoding transcriptional repressors are indispensable for maintenance of stem cell pluripotency [50, 51]. The PcG proteins form multimeric protein complexes to regulate transcription of development-related genes, which are called as polycomb repressive complexes (PRCs) [52]. Currently, two major PRCs have been identified, PRC1 and PRC2, both of which modify chromatin to stably silence transcription at targeted genes [53]. As an E3 ubiquitin ligase, BMI1 works with its partners to catalyze the PcG-dependent ubiquitination of histone H2A in order to modulate transcription [54]. In tumorigenesis, BMI1 plays important roles in promoting cancer stemness, leading to tumor metastasis, recurrence, and drug resistance [55]. Thus, the development of small molecule inhibitors against BMI1 will offer potential opportunities for cancer treatment. PTC-209, as an important inhibitor of BMI1, downregulated BMI1 by reducing mRNA level. PTC-209 exerts inhibitory effects for several cancers, such as breast cancer, non-small cell lung cancer, and acute myeloid leukemia [30, 56, 57]. However, the effect of PTC-209 in anti-HCC is unclear. Here, we found that BMI1 is critical in constructing the risk score model. Our further studies show that BMI1 is upregulated in HCC tissues and the upregulation of BMI1 is associated with poor outcomes of HCC patients, confirming that BMI1 plays important roles in hepatocarcinogenesis. Importantly, we found that PTC-209 significantly inhibits HCC cell growth and proliferation. Thus, our results identify the inhibition of BMI1 as a potential strategy for HCC treatment. We also analyzed the downstream targets of BMI1 by comparing expression profiles of BMI1 wild-type and BMI1-knockout tissues. Strikingly, we found that these targets are enriched in immune-related events. Moreover, the expression of BMI1 correlates with immune cells' infiltration level in HCC, suggesting that BMI1 might be a novel target for improving HCC immune therapy.

Recently, the proteolysis targeting chimeras (PROTACs) technology attracts growing attention of scientific institutes and pharmaceutical companies [58]. PROTACs are designed based on the ubiquitin-proteasome system to induce the degradation of targeted protein. Briefly, the ligands in PROTACs combine with E3 ligase and the targeted protein, respectively, and the linker connects the two ligands and pulls them closer together. PROTACs show positive results for degrading the “undruggable” oncoproteins which lack of binding pockets by small molecule inhibitors [59]. The PROTACs-related new drugs are tested in clinical trials for cancer therapy [59]. Due to the importance of RNF protein-related ubiquitination in tumorigenesis, it is possible that the PROTACs based on RNFs could be useful for cancer treatment. This will be further studied in the future.

In addition, some limitations should be addressed in the future to increase the possibility of our risk model in HCC diagnosis. First, our study was a retrospective study based on the public datasets. It will be better to validate our model using data from prospective clinical trials. Second, the detailed molecular mechanisms of the RNF genes in hepatocellular carcinogenesis are not fully understood. Moreover, discoveries of effective drugs for targeting prognostic RNFs will be more helpful for HCC treatment by *in vivo* experiments and clinical trials. In the future study, we will try to address this issue in subsequent studies.

In summary, using a systematic and comprehensive biomarker discovery and validation approach, we uncovered that an RNF-related gene signature could act as a prognostic indicator for evaluating prognosis of HCC patients and guide HCC treatment. We also identified that BMI1 is tightly associated with HCC progression, which might be a new therapeutic target for HCC.

## Data Availability

All data generated or analyzed during this study are included in this article and its supplementary files. The analyzed data during the current study are available from the corresponding authors on reasonable request.

## Supplementary Materials

Supplementary Materials Supplementary Figure S1: univariate Cox regression analysis of OS for each prognosis-associated candidate RNF gene.
