# Supplementary figures and images for: The Systematic Analyses of RING Finger Gene Signature for Predicting the Prognosis of Patients with Hepatocellular Carcinoma
Source: J Oncol. 2022 Sep 26;2022:2466006. doi: 10.1155/2022/2466006 (PMC9529411; doi:10.1155/2022/2466006)

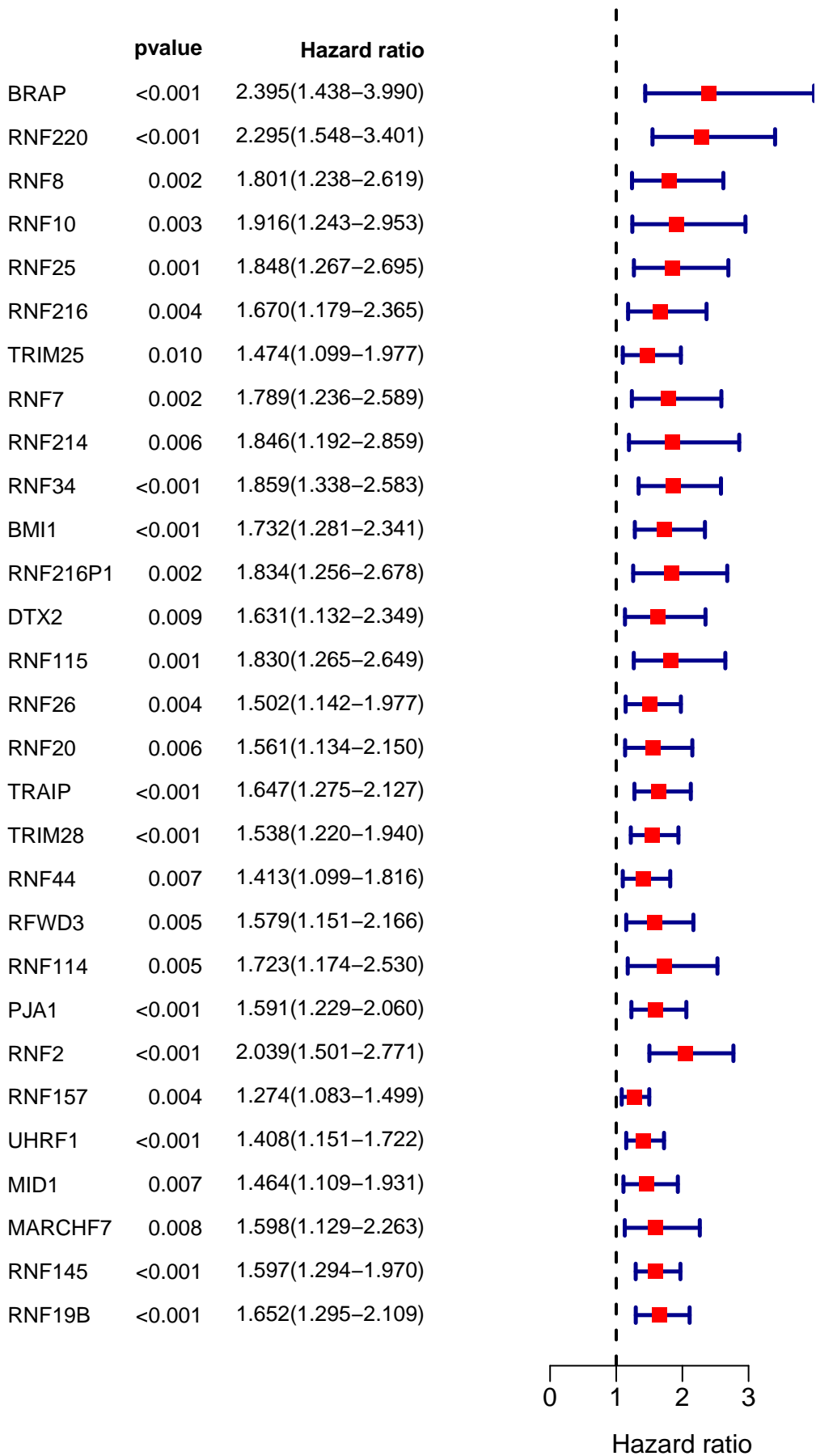

Supplement: Supplementary Materials — Supplementary Figure S1: univariate Cox regression analysis of OS for each prognosis-associated candidate RNF gene. [file 2466006.f1.pdf]
